# Grading of invasive breast carcinoma: the way forward

**DOI:** 10.1007/s00428-021-03141-2

**Published:** 2021-07-01

**Authors:** C. van Dooijeweert, P. J. van Diest, I. O. Ellis

**Affiliations:** 1 Department of Pathology, University Medical Center Utrecht, Utrecht, Netherlands; 2 Department of Internal Medicine, Meander Medical Center, Amersfoort, Netherlands; 3 Department of Histopathology, Nottingham University Hospitals, Nottingham, UK

**Keywords:** Breast, Carcinoma, Histology, Grading

## Abstract

Histologic grading has been a simple and inexpensive method to assess tumor behavior and prognosis of invasive breast cancer grading, thereby identifying patients at risk for adverse outcomes, who may be eligible for (neo)adjuvant therapies. Histologic grading needs to be performed accurately, on properly fixed specimens, and by adequately trained dedicated pathologists that take the time to diligently follow the protocol methodology. In this paper, we review the history of histologic grading, describe the basics of grading, review prognostic value and reproducibility issues, compare performance of grading to gene expression profiles, and discuss how to move forward to improve reproducibility of grading by training, feedback and artificial intelligence algorithms, and special stains to better recognize mitoses. We conclude that histologic grading, when adequately carried out, remains to be of important prognostic value in breast cancer patients.

## History of histologic grading

The importance of the histologic profile of invasive breast cancer in correlation with the disease course was first acknowledged by Von Hansemann in 1893. He presumed that tumors with a loss of differentiation, which he so-called anaplastic, had a greater tendency to metastasize, which he confirmed in 1902. In 1922, MacCarthy and Sistrunk described a correlation between post-mastectomy survival and the degree of differentiation, lymphocytic infiltration, and hyalinization of the tumor [1]. In 1925, Greenough was the first to describe a grading classification system, that, similar to the current classification, separated tumors into three grades of malignancy, based on tubular differentiation, the size of cells/nuclei, and hyperchromatism and mitosis [2]. Several other studies, which also took clinical staging into account, followed. Importantly, it was concluded that histologic grading was of value with regard to prognosis, yet, clinical staging was the most important factor [3].

Until the late 1950s, tumors were simply and only classified according their clinical stage, which does not take into account the accepted range of biological behavior of breast carcinomas. Bloom and Richardson observed at that time that clinical staging provided a useful guide, yet “it fails completely to indicate the likelihood of occult lymphatic and blood-born metastases being present in what appears to be an early case, nor the speed with which such metastases may develop” [4]. This prompted them to develop a technique of histologic grading, which they correlated with survival in a series of 1544 breast cancer patients [4]. In their classification system, tumors were allocated a score of 1–3 for each of three components, differentiation of tubule formation, pleomorphism, and “hyperchromatosis” or mitotic nuclei. A total score, derived from the summation of the three component scores, of 3–5 indicated a low-grade (I) tumor, scores 6–7 an intermediate (II) tumor, and scores of 8–9 a high-grade (III) tumor [4]. Importantly, Bloom and Richardson stated that the different grades were not different pathologic entities, and their 3 grades were based on arbitrarily divisions of a continuous scale of malignancy. They did not claim to have discovered a mathematically accurate grading classification, yet they emphasized that their point system was merely a useful aid in guiding prognosis [4]. Despite these compelling observations, histologic grading of breast cancer was not accepted as a routine procedure, mainly due to perceived reproducibility issues, until decades later [5].

The grading classification of Bloom and Richardson [4] was revised by Elston and Ellis in 1991, who use semiquantitative criteria to improve objectivity and reproducibility [5]. Tubular differentiation was based on evaluation of the percentage of tubule formation, hyperchromatic figures were excluded from assessment, and mitosis was counted using a defined field area. The degree of nuclear pleomorphism was scored according to more objective definitions based on comparison with normal cell types. Elston and Ellis demonstrated the relevance of histological grade in breast cancer and its strong correlation with prognosis, in a series of 1813 patients with primary operable disease who had been followed up for many years [5]. This Elston–Ellis modification of the Bloom and Richardson grading classification (also known as the Nottingham grading system (NGS)) has become globally used to guide management of invasive breast carcinoma [6–8].

## Histologic grading: the basics

Histologic grade represents the degree of differentiation, which reflects the resemblance of tumor cells to normal breast cells. The NGS is a semiquantitative assessment of three morphological characteristics, being tubule/gland formation, nuclear pleomorphism, and mitotic frequency. The NGS is a simple and cheap method, which in principle can be performed in all breast cancer cases [9]. Furthermore, it merely requires appropriately prepared hematoxylin–eosin (HE)-stained tumor slides from optimally formalin-fixed paraffin-embedded tissue blocks by trained experienced pathologists, who are prepared to take the time to diligently follow the standard protocol.

**Fig. 1:**
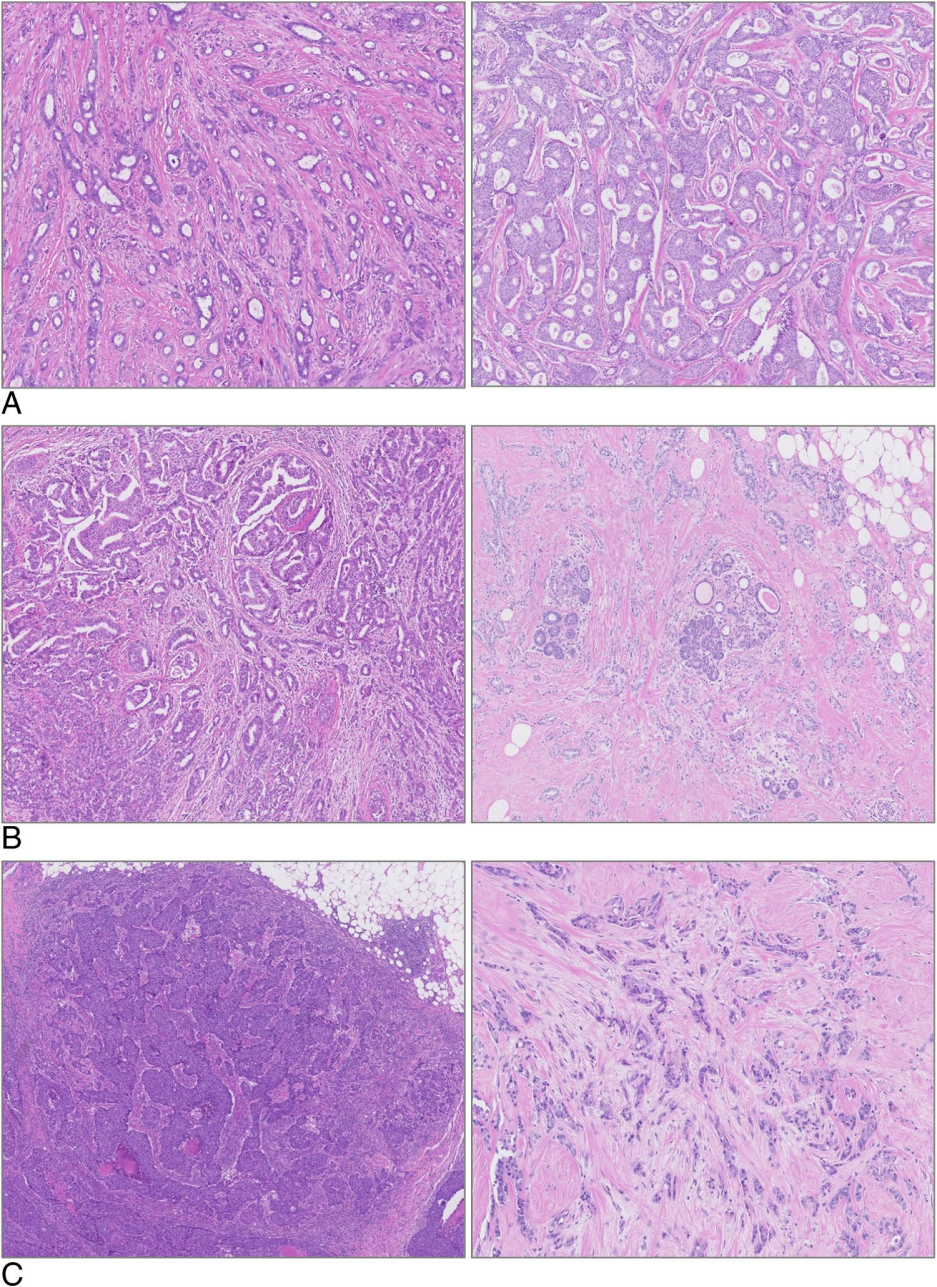
A Tubular differentiation score 1. B Tubular differentiation score 2. C Tubular differentiation score 3

Grading itself is evaluated by a numerical scoring system of 1–3 per category (tubule formation, nuclear pleomorphism, mitotic count). Tubule/gland formation is classified according to the percentage of tubular or glandular acinar spaces (> 75% score 1, 10–75% score 2, < 10% score 3), where only those structures with clear central lumina enclosed by polarized cells are counted (Fig. 1A–C). The inside-out polarization features of micropapillary invasive carcinoma does not by itself count as tubule formation, although these cancers can have tubule formation on the inside of the micropapillary groups that counts as tubules.

**Fig. 2:**
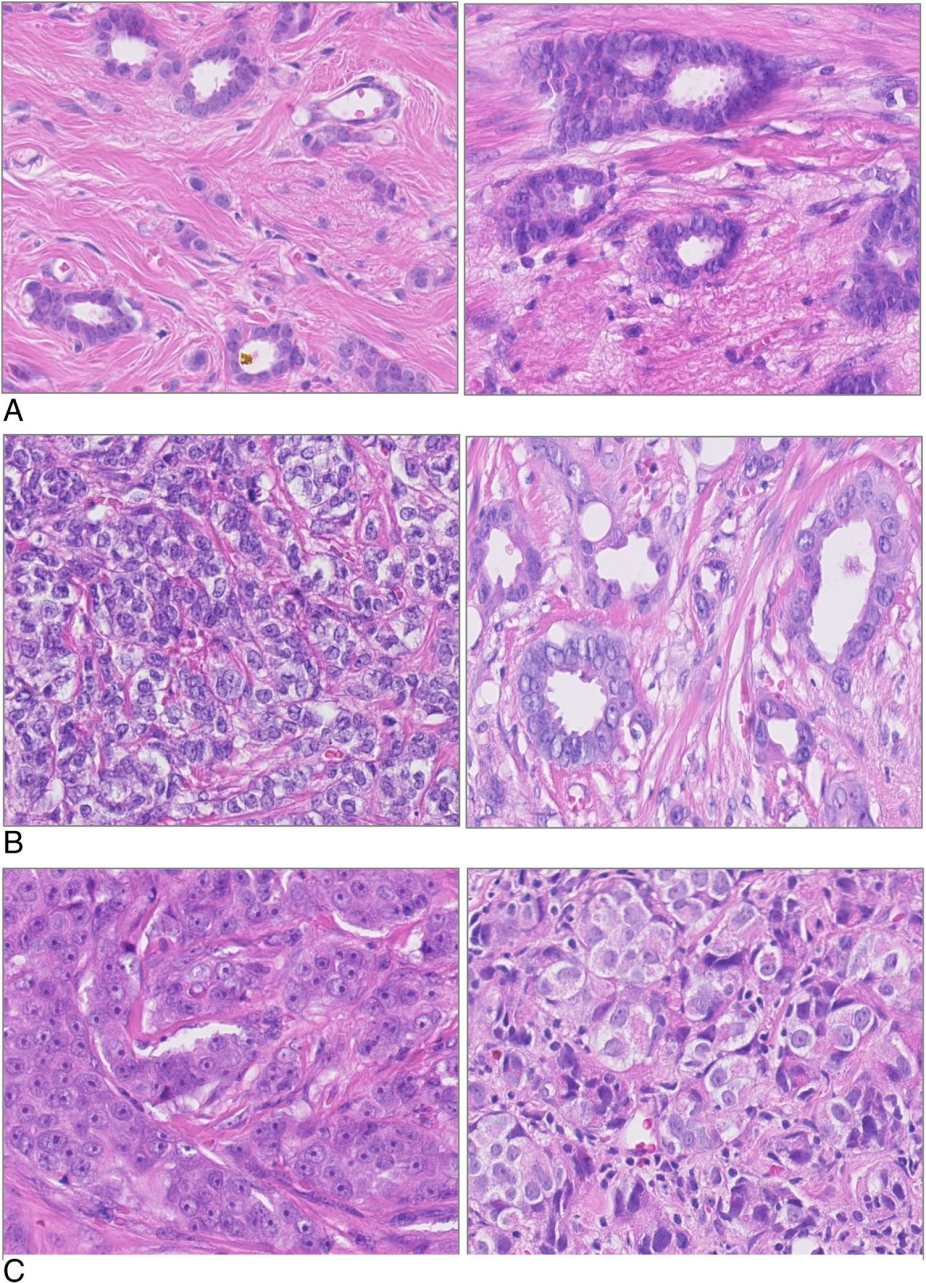
A Nuclear pleomorphism score 1. B Nuclear pleomorphism score 2. C Nuclear pleomorphism score 3

Nuclear pleomorphism, describing the size and degree of variation in tumor cell nuclear size and shape, is scored in the least differentiated area of the tumor. It is assessed by examining the regularity of nuclear size and compared to the shape of normal epithelial cells in the surrounding tissue. Score 1 is allocated to tumor cells that are similar in size to normal epithelial cells, which show only minimal pleomorphism and whose nucleoli and chromatin pattern are inconspicuous at most (Fig. 2A). Tumor cells with nuclei that are 1.5–2 × larger than epithelial cells and with moderate pleomorphism and still inconspicuous nucleoli are given score 2 (Fig. 2B). In contrast, score 3 nuclei are more than 2 × larger in size, which vary considerably in size and which show vesicular chromatin and often prominent nucleoli (Fig. 2C).

**Fig. 3:**
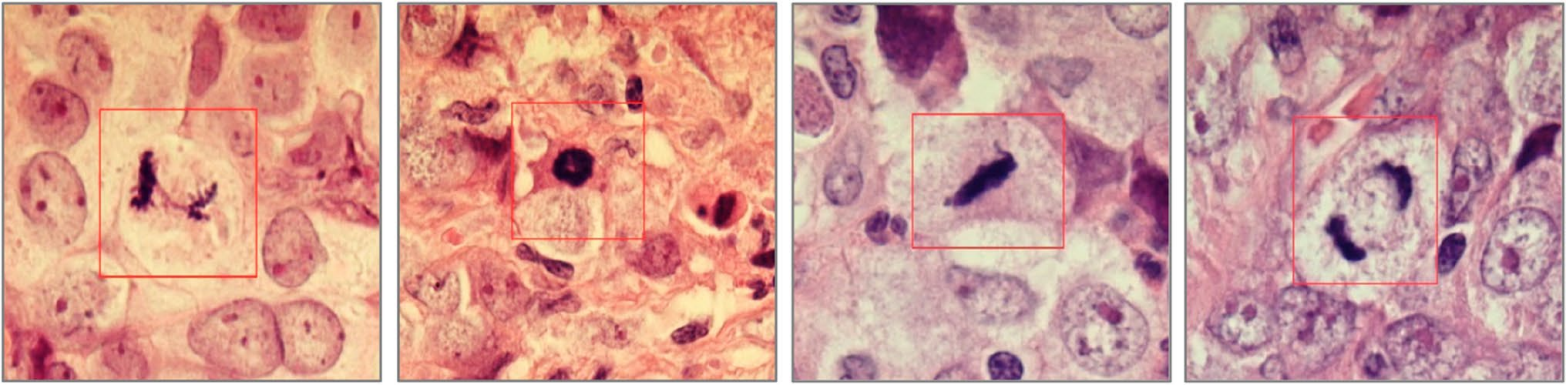
Mitotic figures

Mitotic counting is performed in the most proliferative area of the tumor, usually the most solid area, typically at the periphery of the tumor. A score of 1–3 is based on the number of defined mitotic figures seen in a given tumor area or microscope field area, with cutoff points dependent on field area size assessed using the diameter of the high-power-field (HPF) (Table 1). Examples of well-defined mitotic figures can be found in Fig. 3.

**Table 1:** Score thresholds for mitotic counts

Field	Field	Mitotic count (score)		
Diameter (mm)	Area (mm^2^)	1	2	3
0.40	0.126	≤ 4	5–9	≥ 10
0.41	0.123	≤ 4	5–9	≥ 10
0.42	0.138	≤ 5	6–10	≥ 11
0.43	0.145	≤ 5	6–10	≥ 11
0.44	0.152	≤ 5	6–11	≥ 12
0.45	0.159	≤ 5	6–11	≥ 12
0.46	0.166	≤ 6	7–12	≥ 13
0.47	0.173	≤ 6	7–12	≥ 13
0.48	0.181	≤ 6	7–13	≥ 14
0.49	0.188	≤ 6	7–13	≥ 14
0.50	0.196	≤ 7	8–14	≥ 15
0.51	0.204	≤ 7	8–14	≥ 15
0.52	0.212	≤ 7	8–15	≥ 16
0.53	0.221	≤ 8	9–16	≥ 17
0.54	0.229	≤ 8	9–16	≥ 17
0.55	0.237	≤ 8	9–17	≥ 18
0.56	0.246	≤ 8	9–17	≥ 18
0.57	0.255	≤ 9	10–18	≥ 19
0.58	0.264	≤ 9	10–19	≥ 20
0.59	0.273	≤ 9	10–19	≥ 20
0.60	0.283	≤ 10	11–20	≥ 21
0.61	0.292	≤ 10	11–21	≥ 22
0.62	0.302	≤ 11	12–22	≥ 23
0.63	0.312	≤ 11	12–22	≥ 23
0.64	0.322	≤ 11	12–23	≥ 24
0.65	0.332	≤ 12	13–24	≥ 25
0.66	0.342	≤ 12	13–24	≥ 25
0.67	0.352	≤ 12	13–25	≥ 26
0.68	0.363	≤ 13	14–26	≥ 27
0.69	0.374	≤ 13	14–27	≥ 28

Overall, the three grade component values are summated, resulting in a total score of between 3 and 9 and then categorized into final grade. Scores 3–5 represent well-differentiated grade I tumors, scores 6–7 represent moderately differentiated grade II tumors, and scores 8–9 represent poorly differentiated grade III tumors.

## Histologic grading: prognostic value

The NGS has shown to be of independent significance with regard to breast cancer prognosis [5, 7, 10–12]. In addition, histologic grade has been incorporated in prognostic index scores, of which the Nottingham prognostic index (NPI) [13] is regarded as the only index score that has been extensively validated and which retains its predicting ability in most independent populations [14, 15]. Within the NPI, histologic grade is combined with lymph node (LN) status and tumor size, where grade is considered equally important as lymph node status. In contrast, studies have suggested that histologic grade predicts tumor behavior more accurately than tumor size, which may be considered a more “time-dependent” factor [7, 8, 11, 13, 16].

Breast cancer is now detected at earlier stages by mammographic screening programs, thereby resulting in a greater proportion of both smaller [17, 18] and lymph node–negative tumors at diagnosis [19]. This furthermore increases the clinical contribution of histologic grade [7, 20].

The prognostic significance of histologic grading has been widely studied [5, 7, 10–12, 16, 21–29]. Henson et al. [29] included a spectacularly large number of 22,616 breast cancer cases. They showed similar prognosis for breast cancer patients with stage II/grade I disease and breast cancer patients with stage I/grade II disease [29]. Furthermore, they showed an excellent prognosis for small (< 2 cm) grade I tumors, even when they showed lymph node metastases at presentation. Therefore, Henderson et al. concluded that using histologic grade in conjunction with disease stage (consisting of tumor size and lymph node status) could improve outcome predictions [29].

These results were further supported by a somewhat more recent, retrospective series of 2219 operable breast cancer cases from a single institution by Rakha et al. [7]. Histologic grade proved to be associated with both breast cancer-specific and disease-free survival in the whole series, as well as within the specific subgroups of small tumors (T1a, T1b, T1c) and lymph node–negative and lymph node–positive tumors [7]. The latter has also been shown in other studies [7, 22, 24, 28, 30]. More importantly, the prognostic value of histologic grade was independent of tumor size and lymph node status [7]. Furthermore, it was shown that grade is complimentary and equivalent in impact magnitude to lymph node status, which is widely regarded as a major prognostic factor in breast cancer. For example, patients with grade II tumors and 1–3 positive lymph nodes had a better prognosis than patients with grade III tumors without any lymph node metastases [7]. Moreover, the Swedish two-county trial demonstrated that the independent prognostic effect of histologic grading on survival (as well as lymph node status and tumor size) is long-lasting (> 10 years) [26]. Of note, although grading was initially deemed applicable to Not Otherwise Specified (“ductal”) cancers, grading has been proven prognostically important across all histologic breast cancer types.

In addition, the prognostic role of histologic grading in specific subgroups, for whom the benefit of adjuvant chemotherapy is uncertain, like patients with low-volume lymph node metastases, or patients with ER-positive/lymph node–negative breast cancer, has also been established. For example, histologic grade is an independent prognostic factor in breast cancer patients with ER-positive disease, with [31] or without neoadjuvant endocrine therapy [32]. Furthermore, histologic grade has been shown to be one of the two remaining prognostic factors that was associated with relapse-free survival in a multivariate analyses of ER-positive/HER2-negative breast cancer patients [33].

As to the relative prognostic contribution of the three constituents of grade, several studies have shown that the mitotic count is the most important variable followed by nuclear atypia and then tubule formation [34–36].

Thus, as grade is an important and long-term prognostic factor across breast cancer subtypes, being equally important as lymph node status, more important than tumor size, and being of specific prognostic influence in different subgroups, it would be an omission to exclude histologic grade from clinical decision-making. Fortunately, histologic grade is currently widely incorporated in clinical breast cancer guidelines such as ASCO [37–39], NCCN [40], ESMO [41], UK NICE [42] based on PREDICT [43], and the St. Gallen Expert Panel [44].

## Histologic grading: reproducibility issues

Although histologic grade has long known to be of prognostic value, its reproducibility has also been the subject of debate for decades. Firstly, the distribution of grade varies largely (i.e., up to 27%) between studies (Table 2) [7, 16, 22–24, 26, 27, 30, 35, 45–56]. However, these differences may partly be explained by the wide variety of patient cohorts (Table 2). For example, these cohorts vary in age, type of detection method (screening versus symptomatic, early, or advanced breast cancer), and type of tissue fixation.

**Table 2:** Distribution of histologic grades in different invasive breast cancer studies

Study	Number	Grade 1	Grade 2	Grade 3
Elston, 1984 [45]	625	17%	37%	46%
Davis et al., 1986 [46]	1537	22%	49%	29%
Hopton et al., 1989 [47]	874	29%	46%	25%
Le Doussal, et al., 1989 [35]	1262	11%	45%	46%
Balslev et al., 1994 [48]	9149	32%	49%	19%
Saimura et al., 1999 [22]	741	19%	37%	44%
Reed et al., 2000 [30]	613	25%	41%	35%
Simpson et al., 2000 [24]	368	22%	45%	33%
Lundin et al., 2001 [23]	1554	26%	47%	27%
Frkovic-Grazio and Bracko, 2002 [16]	270	38%	38%	24%
Warwick et al., 2004 [26]	1988	23%	37%	40%
Williams et al., 2006 [49]	1058	20%	46%	34%
Rakha et al., 2008 [7]	2219	18%	36%	46%
Thomas et al., 2009 [50]	1650	26%	45%	29%
Blamey et al., 2010 [27]	16,944	29%	41%	30%
Puig-Vives et al., 2013 [51]	2122	20%	47%	33%
Seneviratne et al., 2015 [52]	2146	25%	52%	23%
Sun et al., 2015 [53]	1259	18%	62%	20%
Moller et al., 2016 [54]	81,427	16%	52%	32%
Van Dooijeweert et al., 2019 [55]	33,792	28%	48%	24%
Van Dooijeweert et al., 2020 [56]	17,102	31%	49%	19%

Secondly, inter- and intra-observer variation has been extensively reported, with a wide range Kappa values (0.43 and 0.85) which correlates to a range from “fair” to “almost perfect” agreement [9, 12, 25, 30, 57–69] (Table 3). A recent nationwide study in the Netherlands showed substantial variation in grading in daily clinical practice, both between pathology laboratories and between pathologists within individual laboratories [55]. Importantly, these differences were not explained by differences in case mix [55]. Subsequently, initiatives were launched to improve variation in grading. Feedback reports in which pathologists and laboratories were benchmarked against the nationwide average and their colleagues (all anonymized) were sent [56], and pathologists and residents were trained using e-learning [70]. Both initiatives resulted in a promising decrease in grading variation and may be implemented broadly in the field of pathology. Yet, it remains important to emphasize what Bloom and Richardson rightfully stated in 1957. The different grades are not different entities, they are not a fact, nor the truth, but they are (statistically) computed cutoff values in a spectrum of histopathologic features on a continuous scale of malignancy. With the clinical implications in mind [20], educating clinicians (like medical oncologists, radiotherapists, surgeons), who use histologic grade as an absolute cutoff, may be equally important to the improvement of histologic grading by pathologists. Furthermore, in the current era of shared decision-making, patients should perhaps be involved as well.

**Table 3:** Inter- and intra-observer reproducibility studies on grading of invasive breast cancer

Study	Cases	Readers	Inter-observer variation	Intra-observer variation
Theissig et al., 1990 [12]	166	3	Complete agreement 72.3%. Kappa 0.57	-
Robbins et al., 1995 [57]	50	5	Complete agreement 83.3%. Kappa 0.73	-
Frierson et al., 1995 [58]	75	6	Kappa 0.43 to 0.74	-
Jacquemier et al., 1998 [59]	24	21	Complete agreement 69%. Kappa 0.53	-
Sikka et al., 1999 [60]	40	3	Kappa 0.68 to 0.83	-
Anderson et al., 2000 [25]	52	2	Kappa 0.54	-
Boiesen et al., 2000 [9]	93	7	Kappa 0.54	-
Reed et al., 2000 [30]	613	2	Kappa 0.69	-
Page et al., 2001 [61]	425	2	Complete agreement 76%	-
Meyer et al., 2005 [62]	7	49	Kappa 0.50–0.59	-
Chowdhury et al., 2006 [63]	50	5	Mean polychoric correlation 0.8	-
Longacre et al., 2006 [64]	35	13	Kappa 0.5 to 0.7	-
Ellis et al., 2006 [65]	12	600	Kappa 0.45 to 0.53 (figures after application of guidelines)	-
Bueno-de-Mesquita et al., 2010 [66]	694	2	Kappa 0.56	
Postma et al., 2013 [67]	310	2	Kappa 0.80	
Rabe et al., 2019 [68]	100	6	Kappa 0.58–0.85	Mean Kappa 0.77
Ginter et al., 2020 [69]	143	6	Kappa 0.50	-

## Histologic grading: the way forward

### Immunohistochemistry for proliferation markers

With the proliferation marker mitotic index being the most important constituent of grade, with observer variation in counting [34–36], one could argue that replacing mitotic index with a potentially more objective method based on immunohistochemical staining of proteins highlighting proliferating cells could help to reduce variation. Ki67 and phosphohistone H3 (PHH3) have shown most promise here. Ki67 is a protein expressed in all phases of the cell cycle, except for resting cells in the G0 phase. However, there is controversy with regard to its clinical utility in routine clinical management, due to variation in analytical practice [71–73] and the absence of consensus on cutoff values [74]. PHH3 is highly expressed in cells in the mitotic phase, and this proliferation marker has shown great promise, also with regard to reproducibility [75]. It may help to better identify mitotic cells and highlight the areas of highest proliferation and thereby increase reproducibility, especially in cases with sub-optimal fixation associated compromised morphology. More research on its clinical utility is necessary [75].

Another potentially important tool is the IHC-4 algorithm, which consists of a combination of ER-, PR-, HER2-, and Ki-67 status [76]. However, to date, this system has not been widely incorporated in breast cancer guidelines, mainly due to the above-mentioned lack of consensus on the clinical utility of Ki67-assessment.

### Molecular profiling and gene expression profiling

In the past decade, molecular profiling and gene expression profiling (GEP) have emerged as new tools to predict tumor behavior. Molecular profiling studies showed that grade I, II, and III breast cancers are most likely different entities as they show specific molecular profiles at immunohistochemical, genomic, and transcriptomic levels, which further supports the relevance of histologic grading [77]. Furthermore, histologic grading has been shown to better correspond to the molecular profile of breast cancer than lymph node status and tumor size [78–80].

Although these “new” biomarker/molecular profile methods were launched with great excitement, it seems unlikely that molecular or gene expression profiling will substitute classic clinicopathologic variables. For ER-positive disease, for example, histologic grade remains an independent prognostic factor in multivariate models, even when molecular signatures are included [81, 82]. In addition, several studies have shown that the added value of GEPs to clinicopathologic variables (age, ER-status, lymph node status) in prognostic models may be limited and sometimes only equal to prognostic indices like the NPI [31, 83, 84].

Two well-known GEPs, Oncotype DX [81] and MammaPrint [85], are currently being used in daily clinical practice in some countries. However, it is also important to acknowledge that these tests are not accessible (i.e., $4.000 per Oncotype DX/per MammaPrint) nor applicable to every breast cancer patient [86–94]. Even more so, studies have shown that “simple” biomarkers like histologic grade and progesterone status can predict Oncotype DX scores, thereby saving the need for these expensive GEPs [95-100]. However, GEPs may be of added value in patients for whom the indication for adjuvant therapy remains doubtful based on classic biomarkers [85]. Lastly, molecular signatures and GEPs are not without flaws themselves, although they are generally considered to be more objective biomarkers [82]. For example, in the MammaPrint study, a change in the RNA extraction solution that was used to calculate the MammaPrint score led to a shift of genomic risk scores in > 150 patients [85]. In addition, similar to statistical cut-offs used for histologic grading, molecular tests and GEPs also depend on biostatistical approaches. Furthermore, intratumor heterogeneity has been found to affect the prognostic risk stratification by GEPs in early breast cancer [101]. Lastly, the results of GEPs and molecular profiles also depend on well-prepared tissue samples to begin with. Overall, molecular profiling and GEPs should not be seen as the new “holy grail” and will not substitute but rather complement classic clinicopathologic biomarkers, which in return need to be assessed adequately, by well-trained pathologists.

### Artificial intelligence

Artificial intelligence methodology is currently finding considerable traction as a tool to aide pathologists. It is expected to be especially helpful with regard to reproducibility concerns that surround histologic grading in its current state. An example of this is the CAMELYON 16 challenge, which showed that some deep-learning algorithms achieved better diagnostic performance in detecting lymph node metastases in breast cancer patients than routine pathologists (under time pressure) and comparable diagnostic performance to expert pathologists (without any time constraints) [102]. Since then, promising results have been published, for example, on predicting tumor proliferation in breast cancer by deep-learning (TUPAC16 challenge) [103] and mitosis counting [104]. However, practical utility studies need to be performed [102, 103]. In addition, it is important to acknowledge that well-annotated (consensus based) datasets are required for the development of AI algorithms. A major pathology-led consortium with 46 partners from all fields of research and businesses, which aims to create a platform of Whole Slide Imaging (WSI) data to develop advanced AI algorithms (BIGPICTURE), may be very helpful here and will start in the near future. These advanced AI algorithms may be able to directly grade breast carcinoma themselves [101].

## Conclusions

In conclusion, histologic grading is a simple and inexpensive method to assess tumor behavior and patient prognosis, thereby identifying patients at risk for adverse outcomes, who may be eligible for (neo)adjuvant therapies. However, histologic grading needs to be performed accurately, on properly fixed specimens, and by adequately trained dedicated pathologists that take the time to diligently follow the protocol methodology. Levels of inter-observer variation can and should still be improved. Feedback and training may be helpful tools to support this. In addition, artificial intelligence is very likely to be able to support pathologists in the near future. When accessible to patients, GEPs may complement classic pathology biomarkers in doubtful cases, rather than substitute them. Furthermore, the GEPs are costly and have flaws of their own. Hence, histologic grading, when adequately carried out, remains to be of important prognostic value in breast cancer patients.

## Data Availability

Not applicable.
